# Draft Genome Sequence of *Vibrio owensii* Strain SH-14, Which Causes Shrimp Acute Hepatopancreatic Necrosis Disease

**DOI:** 10.1128/genomeA.01395-15

**Published:** 2015-12-03

**Authors:** Liyuan Liu, Jinzhou Xiao, Xiaoming Xia, Yingjie Pan, Shuling Yan, Yongjie Wang

**Affiliations:** a College of Food Science and Technology, Shanghai Ocean University, Shanghai, China; b Laboratory of Quality and Safety Risk Assessment for Aquatic Products on Storage & Preservation (Shanghai), Ministry of Agriculture, Shanghai, China; c Institute of Biochemistry and Molecular Cell Biology, University of Goettingen, Goettingen, Germany

## Abstract

We sequenced *Vibrio owensii* strain SH-14, which causes serious acute hepatopancreatic necrosis disease (AHPND) in shrimp. Sequence analysis showed a large extrachromosomal plasmid, which encoded *pir* toxin genes and shared highly sequence similarity with the one observed in AHPND-causing *Vibrio parahaemolyticus* strains. The results suggest that this plasmid appears to play an important role in shrimp AHPND.

## GENOME ANNOUNCEMENT

Acute hepatopancreatic necrosis disease (AHPND) is caused by some pathogenic strains of *Vibrio parahaemolyticus* (1). However, a strain of *Vibrio owensii* (strain SH-14) was isolated from the hepatopancreas of diseased *Penaeus vannamei* collected from a shrimp farm in Shanghai, China. It formed mauve-colored colonies when cultured on CHROMagar Vibrio. Based on shrimp bioassays (2) and PCR detection methods (http://www.enaca.org/publications/health/disease-cards/ahpnd-detection-method-announcement.pdf), the *V. owensii* strain SH-14 has the ability to cause AHPND.

The genomic DNA of *V. owensii* strain SH-14 was extracted with a TIANamp Bacteria DNA kit (TIANGEN, Beijing), and sequenced on an Illumina MiSeq sequencer at Majorbio Bio-Pharm Technology Co., Ltd., Shanghai, China. The total clean paired-end reads were 522,671,876 bp (average coverage 108.84×). The genome of *V. owensii* strain SH-14 was assembled into 120 scaffolds (*N*_50_, 426,824 bp) and 69 contigs >1,000 bp using SOAPdenovo v2.04 and GapCloser v1.12 (3). The largest contig was 914,734 bp. The contigs contained 5,388 predicted coding sequences annotated by using Glimmer 3.02 (4), 105 untranslated rRNA sequences annotated by Barrnap 0.4.2 (5), and 4 untranslated tRNA sequences annotated by tRNAscan-SE v1.3.1 (6).

Type IV pilus adherence system and several iron transporter and secretion systems (type II, IV, and VI) were identified. At least four virulence proteins were annotated. Several proteases were found, six of which were zinc-dependent proteases. In addition, bacteriophage related genes were also identified.

A large (69,142 bp) extrachromosomal plasmid was obtained, which shared 99.1% of pairwise identity with the one detected in AHPND-causing *V. parahaemolyticus* strains (7). This plasmid contained 99 open reading frames, which encoded mating pair formation proteins, transposases, type II and III secretion system proteins, and homologues to the insecticidal *Photorhabdus* insect-related binary toxin PirAB (1). The results suggest that the plasmid appears to play an important role in shrimp AHPND.

### Nucleotide sequence accession numbers.

This whole-genome shotgun project has been deposited at DDBJ/EMBL/Embank under the accession no. LKMC00000000. The version described in this paper is version LKMC01000000.
